# Deep Learning With Electronic Health Records for Short-Term Fracture Risk Identification: Crystal Bone Algorithm Development and Validation

**DOI:** 10.2196/22550

**Published:** 2020-10-16

**Authors:** Yasmeen Adar Almog, Angshu Rai, Patrick Zhang, Amanda Moulaison, Ross Powell, Anirban Mishra, Kerry Weinberg, Celeste Hamilton, Mary Oates, Eugene McCloskey, Steven R Cummings

**Affiliations:** 1 Digital Health & Innovation Amgen Inc Thousand Oaks, CA United States; 2 Global Medical Operations Amgen Inc Thousand Oaks, CA United States; 3 US Medical Amgen Inc Thousand Oaks, CA United States; 4 Department of Oncology & Metabolism The University of Sheffield Sheffield United Kingdom; 5 Department of Medicine University of California San Francisco San Francisco, CA United States

**Keywords:** fracture, bone, osteoporosis, low bone mass, prediction, natural language processing, NLP, machine learning, deep learning, artificial intelligence, AI, electronic health record, EHR

## Abstract

**Background:**

Fractures as a result of osteoporosis and low bone mass are common and give rise to significant clinical, personal, and economic burden. Even after a fracture occurs, high fracture risk remains widely underdiagnosed and undertreated. Common fracture risk assessment tools utilize a subset of clinical risk factors for prediction, and often require manual data entry. Furthermore, these tools predict risk over the long term and do not explicitly provide short-term risk estimates necessary to identify patients likely to experience a fracture in the next 1-2 years.

**Objective:**

The goal of this study was to develop and evaluate an algorithm for the identification of patients at risk of fracture in a subsequent 1- to 2-year period. In order to address the aforementioned limitations of current prediction tools, this approach focused on a short-term timeframe, automated data entry, and the use of longitudinal data to inform the predictions.

**Methods:**

Using retrospective electronic health record data from over 1,000,000 patients, we developed Crystal Bone, an algorithm that applies machine learning techniques from natural language processing to the temporal nature of patient histories to generate short-term fracture risk predictions. Similar to how language models predict the next word in a given sentence or the topic of a document, Crystal Bone predicts whether a patient’s future trajectory might contain a fracture event, or whether the signature of the patient’s journey is similar to that of a typical future fracture patient. A holdout set with 192,590 patients was used to validate accuracy. Experimental baseline models and human-level performance were used for comparison.

**Results:**

The model accurately predicted 1- to 2-year fracture risk for patients aged over 50 years (area under the receiver operating characteristics curve [AUROC] 0.81). These algorithms outperformed the experimental baselines (AUROC 0.67) and showed meaningful improvements when compared to retrospective approximation of human-level performance by correctly identifying 9649 of 13,765 (70%) at-risk patients who did not receive any preventative bone-health-related medical interventions from their physicians.

**Conclusions:**

These findings indicate that it is possible to use a patient’s unique medical history as it changes over time to predict the risk of short-term fracture. Validating and applying such a tool within the health care system could enable automated and widespread prediction of this risk and may help with identification of patients at very high risk of fracture.

## Introduction

Fractures due to osteoporosis and low bone mass are associated with a significant personal, clinical, and economic burden. These fractures are common; the risk of sustaining such a fracture increases with age, and their incidence is expected to increase worldwide as the population ages [1-11]. In the United States, an estimated 1 in 2 women and 1 in 4 men over 50 years of age will experience such a fracture [12-14]. However, there remains a significant diagnosis and treatment gap for osteoporosis [1,2,4,12]. When these fractures occur, they often result in a loss of independence for patients and can lead to functional disability, lower quality of life, and increased mortality [5,15-38]. Given this substantial burden and unmet need for interventions, it is critical to identify patients at risk of fracture, as effective management of risk can prevent these deleterious outcomes.

Several fracture risk prediction tools have been developed for clinical use. The most commonly used tools are the University of Sheffield Fracture Risk Assessment Tool, known as FRAX [39], and the Garvan Institute of Health Bone Fracture Risk Calculator (GIH-BFRC) [40]. Both tools use a set of cross-sectional clinical risk factors to evaluate fracture likelihood, and typically require manual data entry to perform the predictions. The performance of both methods varies greatly in real-world analyses; this variance is partially explained by study population and design and predicted fracture outcome (hip vs other osteoporotic fractures). In a review [41], 12 studies of FRAX showed an average area under the receiver operating characteristics curve (AUROC) of 0.65 (SD 0.038) when predicting major osteoporotic fractures without including bone mineral density in the model, and similar results were shown for GIH-BFRC [41]. These commonly used risk assessment tools estimate 5- and 10-year fracture risk but do not provide estimates of 1- to 2-year risk [42-45].

Increased risk of fracture in the next 1-2 years is not routinely assessed in clinical practice, despite the existence of rapid-acting preventative therapeutics [8,46,47]. Although methods for predicting short-term risk have been explored [48-50], they have not yet been widely clinically accepted. Furthermore, these models are limited to a specific set of cross-sectional information, some of which may not readily be available. Thus, there remains a need to further develop a fracture risk prediction tool that predicts on a short-term time frame in order to facilitate identification of patients at high risk. While there are published examples [51-53] applying artificial intelligence to fracture and osteoporosis risk, these approaches focus either on imaging data [51] or on cross-sectional data for long-term predictions [52,53]. To our knowledge, there is no existing method that applies deep learning to sequential patient data for predicting fracture risk.

To address these unmet needs, we developed *Crystal Bone*, a machine learning approach that leverages techniques typically applied in natural language processing. However, rather than applying these methods to text-based data, we applied them to longitudinal data contained in electronic health records. Specifically, we focused on diagnosis codes (International Classification of Diseases; ICD), treating each code as a word and sequences of codes as stories. The goal of this study was to evaluate the ability of these natural language processing–based models to learn patterns associated with increased short-term (ie, 2-year) fracture risk. The results of our analyses suggest that not only does this unique longitudinal method produce accurate short-term fracture risk predictions, but also that the approach can help fulfill the unmet need that exists in fracture-risk identification.

## Methods

### Data Background

We used subsets of the Optum deidentified electronic health record data set, which contains comprehensive longitudinal electronic health record data for 91 million patients from over 140,000 providers (as of March 2018) from the United States. The subsets, which contain bone health and pan-therapeutic populations respectively, cover the time from January 1, 2007, through December 31, 2018 (Optum, email communication, August 2019).

The bone health subset was obtained by filtering for patients with osteoporosis, fractures, or bone-related medications (n=6,329,986). In the period covered by the data set, the fracture incidence rate (ie, the proportion of fractures among all events detected, which may include multiple fractures per person) was 39% in the population over 50 years of age. The bone health data set was primarily used for training the model.

The pan-therapeutic data set represented a random sample of 5% of the overall Optum electronic health record data set and contained patient data (n=3,476,219) with no filtering for any specific comorbidities or treatments; this dataset had a fracture incidence rate of 8.5% in the population over 50 years of age. Because the sample was drawn from such a large population, the pan-therapeutic data set was assumed to be broadly representative of the US population. As such, we performed all model evaluations on a testing sample from this data set (a holdout data set), to better understand the generalizability of the model in a real-world setting.

### Ethical Approval

Since this was a retrospective study using deidentified data, patients were not required to actively participate in the study. Therefore, neither informed consent of patients nor institutional review board approval was required.

### Data Engineering and Cohort Selection

The cohort consisted of patients who were at least 50 years of age at the time of their event; this criterion was chosen to reduce the data to a population that is more susceptible to fractures associated with osteoporosis and low bone mass. For fracture patients, an event is the date of occurrence of any qualifying fracture. Qualifying fractures are defined by a set of rules based on those used by Wright et al [54] for identifying novel and relevant fracture events in claims data. For nonfracture patients, an event is the date of the last recorded diagnosis of any kind in the data set. We describe further details of the fracture identification process in Multimedia Appendix 1.

We further filtered our cohorts for patients with at least 2 years of medical history leading up to their respective events. Applying these parameters limited the bone health cohort to 3,408,494 patients and the pan-therapeutic cohort to 700,315 patients.

**Figure 1:**
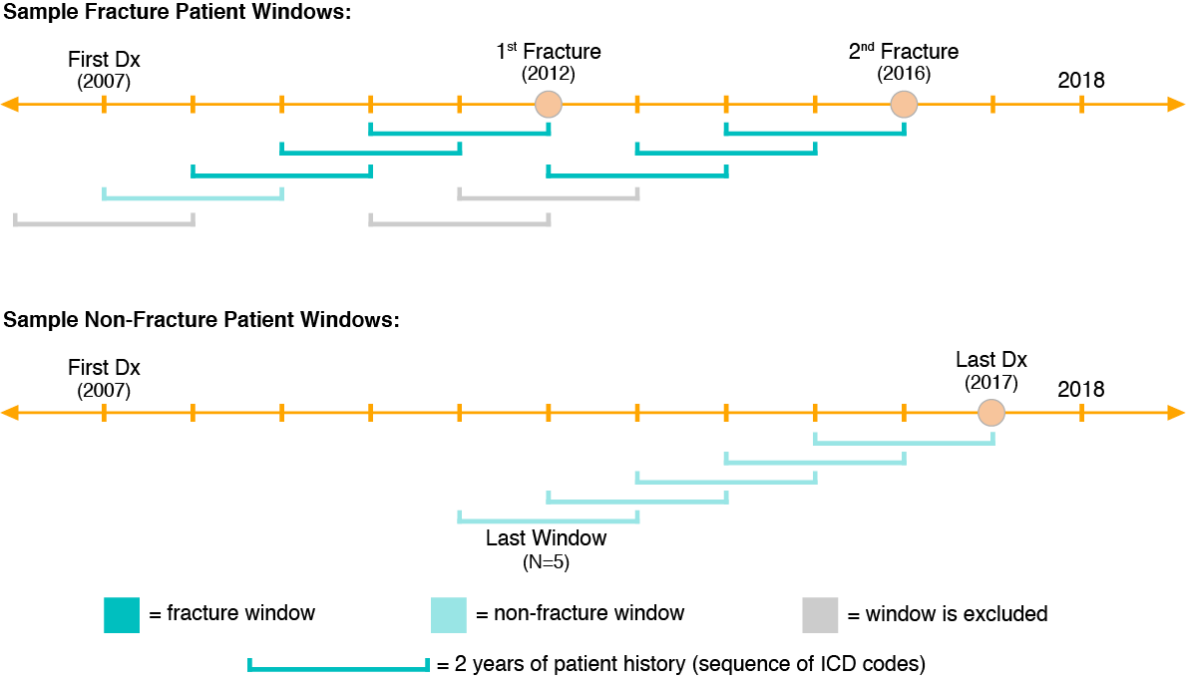
Sliding window algorithm schematic. This schematic depicts the sliding window algorithm for a multifracture and nonfracture patient. Dx:diagnosis; ICD: International Classification of Diseases.

We applied sliding windows to the data (Figure 1), where each event could have up to 5 windows, and each window was a historical sequence defined as the list of chronologically ordered ICD codes in the 2 years leading up to an event. These historical sequences were then used to predict risk of fracture within a 2-year horizon (a 1-year horizon was also explored, see Multimedia Appendix 1). As shown in Figure 1, some windows were dropped from the analysis due to incomplete or potentially overlapping coverage. Additionally, windows that occur more than 2 years before a fracture event were labeled as nonfracture windows. The motivation for this approach was to provide the algorithm with multiple unique code sequences leading up to the same event that may reflect changes in risk at various times within the given time horizon. Furthermore, the fixed window size provided a consistent timeframe for prediction as opposed to varying lengths of time for each patient, which would have occurred if patients’ complete code histories were used. Further details regarding the motivation and methodology of this approach are in Multimedia Appendix 1.

There was no additional filtering based on specific diagnoses or comorbidities. For each qualifying patient, the algorithms utilized all available ICD codes in the historical sequences described above. Only the codes that occurred fewer than 5 times in the full cohort were excluded, as these codes were too rare to be included in the diagnosis code vocabulary.

### Data Sampling

Before model training, we generated a 70:30 random split of the pan-therapeutic data, representing training and holdout subsets. Since the pan-therapeutic data set is highly imbalanced, with a fracture event incidence of only 6.5% after applying the sliding window algorithm, we oversampled additional fracture windows from the bone health data set to achieve a balanced (50:50) training set for modeling. This oversampling training paradigm was replicated for all models. The holdout set remained untouched, with the original distribution of fractures.

### Modeling Approaches

#### Overview

Crystal Bone was inspired by techniques that are typically applied in natural language processing. However, instead of applying these techniques to text-based data, we applied them to sequences of ICD codes. Correspondingly, each ICD code was analogous to a word, and each sequence of ICD codes was analogous to a document. To this end, we implemented 2 distinct frameworks: (1) ICD code vectorization and long short-term memory networks, and (2) patient-level vectorization and extreme gradient boosting decision trees. Both approaches utilize sequences of ICD codes as inputs. The ICD code vectorization and long short-term memory framework undertakes this task by first learning semantic definitions for the codes, then evaluating the sequence of definitions through a deep learning network.. The patient-level vectorization and extreme gradient boosting modeling framework employs a similar approach; however, rather than embedding individual ICD codes, it embeds the entire ICD code sequence for each patient, thereby learning “summaries” of patient sequences. This framework produces a prediction by feeding these summaries through a decision tree classifier. The model parameters were tuned to optimize AUROC; details of this process are provided in Multimedia Appendix 1.

#### Framework 1: ICD Code Vectorization + Long Short-Term Memory

**Figure 2:**
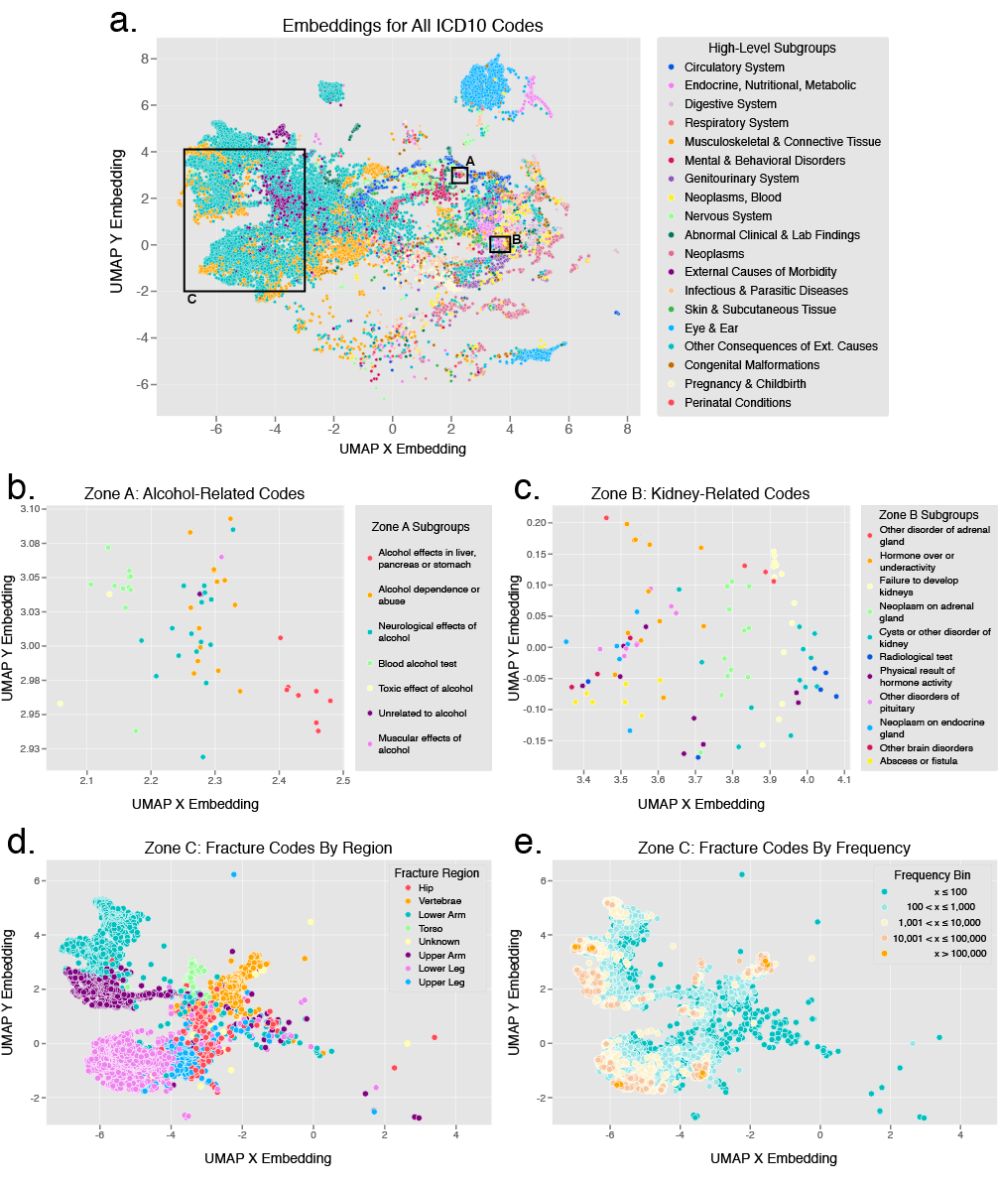
2D projection of ICD-10 code embeddings from the ICD code vectorization model: (a) All ICD-10 codes by the first letter (high-level category) of the code, (b) a cluster of codes related to alcohol near coordinates (2.3, 3) by code subgroups, (c) a cluster of codes related to kidney function near coordinates (3.75, 0.025) by code subgroups, and all ICD-10 fracture codes in region C (d) by region of the body, and (e) by frequency of occurrence. ICD: International Classification of Diseases; UMAP: uniform manifold approximation and projection.

The first framework consisted of 2 primary components. The ICD code vectorization component was responsible for learning a “definition” for each ICD code based on skip-gram architecture word embedding (word2vec) [55], an unsupervised learning approach that mapped each code in the vocabulary to a 100-dimensional vector. To generate these embeddings, we utilized sequences from the pan-therapeutic training set alone (without oversampling), to avoid bias toward bone-health related codes. In our implementation, the vocabulary consisted of all diagnosis codes that occurred at least 5 times in this data set, amounting to more than 40,000 unique codes. The method generated a vector for each code based on the context in which it appeared; in electronic health records, similar ICD codes appear in similar contexts, and as a result have similar vector representations. These embeddings reduced the dimensionality and sparsity of the feature space, and helped the neural network recognize related ICD codes. Figure 2 illustrates the encoded vectors projected onto a 2D space using uniform manifold approximation and projection (UMAP) for dimension reduction [56]. The collocation of related diagnosis codes in this coordinate space provided qualitative evidence that the ICD code vectorization had encoded meaningful latent information.

The long short-term memory component consisted of a neural network with long short-term memory layers, a deep learning architecture that enables the evaluation of recurrent data, such as sequences of embedded ICD codes. We trained this network with the complete training set (including oversampling from the bone health data set). The long short-term memory network predicted the likelihood of a fracture event within 2 years as a classification problem. Long short-term memory networks are a common approach for solving such problems [57].

**Figure 3:**
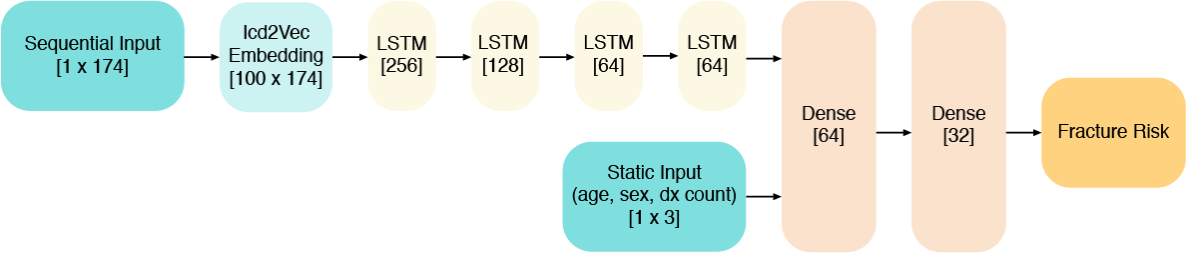
High-level architecture of the long short-term memory neural network including the dimensionality of the inputs, as well as the number of nodes in each layer. Dx: diagnosis; Icd2vec: ICD code vectorization; LSTM: long short-term memory.

Additionally, given the ubiquitous use of nonsequential features such as age and sex for predicting fracture risk, we supplied age and sex to the neural network as static features through concatenation of long short-term memory and dense layers. Furthermore, because the long short-term memory framework required all input sequences to have uniform length, we also included total diagnosis count as a static feature to account for the effects of truncating or padding the sequences. The schematic in Figure 3 provides an overview of the model architecture and inputs to the algorithm, namely age, sex, diagnosis count, and the patient’s unique sequence of ICD codes.

#### Framework 2: Patient-Level Vectorization and Extreme Gradient Boosting

Similar to the ICD code vectorization + long short-term memory modeling framework, the patient-level vectorization and extreme gradient boosting decision trees framework consists of 2 components. First, the patient-level vectorization embeds entire ICD code sequences to a 128-dimensional semantic space using the distributed bag of words framework [58]. Much as the ICD code vectorization learned definitions of individual ICD codes, the patient-level vectorization instead learned summaries of patient sequences. The method for doing so is the same; patients with similar sequential contexts will have similar summary vectors. We trained the patient-level vectorization with the sliding window ICD code sequences, again only utilizing the pan-therapeutic data to avoid bias toward the bone health therapeutic area. This created embeddings that represented 2-year episodes of patient histories; a detailed exploration of these embeddings is in Multimedia Appendix 1.

The extreme gradient boosting decision trees component utilized the embeddings from the patient-level vectorization, as well as the static features of age, sex, and total diagnosis count that were incorporated in Framework 1, to predict fracture risk. This type of algorithm, also referred to as XGBoost, is a scalable tree-based modeling approach that improves the generalizability, speed, and efficacy of prediction [59]. We trained this algorithm with the full training set (including bone health data set oversampling) to learn a classification model that predicted the likelihood of fracture within 2 years.

#### Ensemble Model

An ensemble model was also evaluated. This algorithm combined the outputs of both the aforementioned frameworks with a logistic regression metaclassifier.

#### Baseline Models

We compared these modeling frameworks to 2 baseline models. The first baseline model utilized the age and sex of each patient at each window. These were 2 of the only features shared by the FRAX tool and the GIH-BFRC models. The other shared feature is prior fracture; however, because neither the FRAX tool nor GIH-BFRC’s method of measuring this value was possible for our data set without censoring, we did not include it in the model. The second baseline incorporated age, sex, and total diagnosis count (number of ICD codes) in each sample; these represent all of the static features used by both modelling frameworks, enabling evaluation of the relative benefit of including sequential ICD code data. Both baseline models utilized extreme gradient boosting decision tree algorithms, the same classification approach that was used in Framework 2.

#### Human-Level Performance Approximation

In addition to these baselines, we approximated human-level performance by isolating a set of retrospective physician-prescribed interventions that were identifiable in the electronic health record data set. These interventions consisted of diagnostic tests as well as pharmacologic treatments. The list of interventions was based on treatment guidelines provided by the National Osteoporosis Foundation [60] and the Journal of Clinical Endocrinology and Metabolism [61] and was further validated by the physician coauthors of this manuscript, who confirmed that the interventions aligned with their understanding of osteoporosis treatment guidelines (Table 1). If a patient received one of these interventions in a 2-year historical window, that window was flagged as “physician-identified risk, worthy of intervention.” A full description of the limitations of this approach is described in Multimedia Appendix 1.

**Table 1:** List of physician interventions for human-level performance analysis.

Type and name			Pharmacologic
**Procedure**			
	Dual-energy x-ray absorptiometry	No	
	Vertebral fracture assessment	No	
	Quantitative computed tomography	No	
	Other bone density measurements (single energy x-ray absorptiometry, radiographic absorptiometry, ultrasound, single-photon absorptiometry)	No	
	Bone turnover markers	No	
	Administration of any medications referenced below	Yes	
**Treatment**			
	Bisphosphonates (alendronate, alendronate-cholecalciferol, ibandronate, risedronate, zoledronic acid)	Yes	
	Abaloparatide	Yes	
	Denosumab	Yes	
	Raloxifene	Yes	
	Bazedoxifene	Yes	
	Romosozumab	Yes	
	Teriparatide	Yes	
	Calcitonin	Yes	
**Diagnosis**			
	Osteoporosis (M80, M81, 733.0)	No	

We defined the cohort of patients who did not receive any form of intervention (diagnoses, tests, or treatments) as *no intervention* and assessed how well the algorithm was able to correctly identify which patients had a fracture within 2 years, as well as how frequently the algorithm mistakenly flagged patients with no imminent fracture. We also evaluated the patients who received interventions (the *intervention* cohort) with this method, referred to as the *cohort analysis*. However, since an intervention can directly modulate fracture risk, we performed a separate analysis in order to mitigate some of the uncertainty due to the effects of interventions. For this analysis, we identified each patient’s first pharmacologic intervention and used the diagnosis history leading up to this date as input. This analysis allowed us to gauge the extent to which the algorithm flags agreed with human-level performance interventions (without needing to adjust for their effects). We termed this the *overlap analysis*. The cohort analysis utilized the full list of interventions, while the overlap analysis utilized the pharmacological subset of the list of interventions.

### Model Performance

We report model performance on a set of 5 primary metrics: AUROC, recall (sensitivity), specificity, precision, and area under the precision-recall curve (AUPRC).

## Results

### Model Performance

The overall performance of the algorithms is shown through comparison of the 2 frameworks with the 2 baseline models to demonstrate the quality of each algorithm's predictions. Table 2 shows a summary of key model performance metrics on the same holdout data set. The Crystal Bone models, including the ensemble model that combined the 2 approaches, outperformed the baseline models for nearly all performance metrics.

**Table 2:** Comparison of model performance metrics.

Model	AUROC^a^	Recall	Specificity	Precision	AUPRC^b^
ICD code vectorization + LSTM^c^	0.812	0.646	0.812	0.192	0.462
Patient level vectorization + XGBoost^d^	0.790	0.670	0.758	0.161	0.358
Ensemble	0.818	0.693	0.777	0.177	0.463
Baseline (age, sex)	0.667	0.787	0.416	0.0855	0.119
Baseline (age, sex, diagnosis count)	0.668	0.547	0.707	0.114	0.130

^a^AUROC: area under the receiver operating characteristics curve.

^b^AUPRC: area under the precision-recall curve.

^c^LSTM: long short-term memory.

^d^XGBoost: extreme gradient boosting.

### ICD Code Vectorization + Long Short-Term Memory Model

To further characterize this performance, we evaluated the ICD code vectorization and long short-term memory model on primary and subsequent fracture events. While the model performs best on subsequent fractures, both primary and subsequent fracture analyses (AUROC 0.742 and 0.910, respectively) show a marked improvement against corresponding baseline models (AUROC 0.591 and 0.747, respectively). We report detailed results of this experiment and additional evaluations of sensitivity and robustness of this model in Multimedia Appendix 1.

### Human-Level Performance Comparison

Table 3 contains the results of the cohort analysis. For windows with no interventions, Crystal Bone Framework 1 correctly flagged 16,127 of the 28,626 windows that resulted in fracture (56.3%); this corresponds to 9649 out of 13,765 (70.1%) of the unique fracture events. Crystal Bone Framework 1 incorrectly flagged 91,717 of the 532,621 windows with no fractures as at-risk (17.2%); however, 1053 of the windows in this cohort (3%) sustained a fracture in >2 years.

For windows with interventions, only 11,833 of 69,198 (17.1%) of the detected interventions included treatments; thus, the remaining 57,365 (82.9%) interventions were either diagnoses or diagnostic tests. In the intervention cohort, Crystal Bone Framework 1 correctly captured 10,277 out of 12,244 windows for which fracture occurred within 2 years (83.9%). For the windows with interventions and no fracture event, 19,235 out of 56,954 (33.8%) are incorrectly flagged by our algorithm as at risk. These results suggest Crystal Bone’s ability to recognize interventions through their associated ICD codes and adjust the predicted fracture risk accordingly. However, a deeper exploration of specific interventions is required to verify this.

**Table 3:** Human-level performance results.

Cohort			Windows, n (%)		Flag, n (%)		No flag, n (%)	
**Total**			630,445 (100)		—^a^		—	
	**No intervention**			561,247 (89.0)		—		—
		Fracture	28,626 (5.1)		16,127 (56.3)		12,449 (43.7)	
		Nonfracture	532,621 (94.9)		91,717 (17.2)		440,904 (82.8)	
	**Intervention**			69,198 (11.0)		—		—
		Fracture	12,244 (17.7)		10,277 (83.9)		1967 (16.1)	
		Nonfracture	56,954 (82.3)		19,235 (33.8)		37,719 (66.2)	

^a^Not reported.

The overlap analysis enabled us to better understand how well Crystal Bone Framework 1 correlated with observed physician interventions through exploration of the first pharmacological treatment in the holdout set. Of the 7127 patients who received treatment, 6071 had enough medical history leading up to this treatment for Crystal Bone Framework 1. When evaluating these patients, 3017 out of those 6071 (49.7%) were considered at risk of fracture in 2 years.

We evaluated the incidence of fracture within 2 years for this subgroup. Of the cohort deemed at risk by the algorithm, 684 out of 3017 (22.7%) experienced a fracture within 2 years of the first intervention date. This precision is a slight improvement over that of the algorithm on the overall holdout set, at 19.2%. Furthermore, of all 570 patients in this pharmacological intervention cohort who ultimately suffered from a fracture within 2 years, Crystal Bone Framework 1 correctly flagged 469 (82.3%).

## Discussion

### General

In this study, we evaluated the performance of 2 natural language processing–inspired fracture prediction models: (1) ICD code vectorization and long short-term memory (AUROC 0.812) and (2) patient-level vectorization and extreme gradient boosting (AUROC 0.790). The performance of these models reflected a substantial improvement over 2 baseline models: (1) with age and sex (AUROC 0.670) and (2) with age, sex, total diagnosis count (AUROC 0.670). Furthermore, these short-term prediction metrics were an improvement over cross-sectional tools for long-term time frames, such as FRAX and GIH-BFRC, which have been widely clinically accepted [41]. Although fundamental differences in study design make it impossible to compare these metrics directly, sensitivity analyses of Crystal Bone across fracture types, prediction time frames, and fracture definitions suggest robust predictive performance and generalizability. To our knowledge, this is the first study that has experimented with separate models for primary and subsequent fracture types; further discussion of this analysis, as well as the additional sensitivity analyses, is in Multimedia Appendix 1.

The human-level performance comparison provides deeper insight to the benefits of Crystal Bone. The retrospective labeling utilized in both the cohort and overlap analyses enabled a scalable, data-driven comparison of physician action and Crystal Bone and avoided bias that may occur through alternative methods of human-level performance evaluation [62]. To our knowledge, this is the first fracture risk prediction study which includes such a human-level performance comparison in the analysis.

Through the cohort analysis we learned that only a small proportion of patients received preventative interventions, including basic diagnostic tests, showcasing the extent of unmet need in the health care system [1,2,4,12]. In the subset of patient windows with no interventions, Crystal Bone was able to flag 70.1% of the unique fracture events. Given the existence of rapid-acting preventative therapeutics [8,46,47], as well as the demonstrated efficacy of bone-forming agents in reduction of 1- to 2-year fracture risk [63-69], these results suggest that, had appropriate preventative measures been taken, the risk of these fractures may have been reduced, thus mitigating a significant burden to both the patient and the health care system.

The findings of the overlap analysis further support the merits of Crystal Bone, through demonstration of alignment with observable interventions made by physicians. Because it is impossible to confirm whether these treatment interventions were taken in response to a perceived short-term risk of fracture, we cannot expect 100% overlap between Crystal Bone and these observed interventions. We saw that Crystal Bone was aligned with these physician interventions 49.7% of the time. While this overlap is not complete, it captured 82.3% of the patients who ultimately experienced a fracture, reflecting the algorithm’s increased sensitivity for the cohort deemed at-risk by physicians. This suggests a meaningful alignment with both physician evaluation and actual observed fracture risk. Ultimately, these human-level performance comparisons, coupled with performance against baseline models and alternative risk prediction methods, suggest that Crystal Bone can fulfill a critical unmet need through identification of patients at high risk of fracture.

### Limitations of the Current Approach

Various limitations exist for the approaches described, particularly from the inherent complications of using real-world data. The techniques described rely upon ICD codes recorded in electronic health record systems, which will impact the performance and validity of the models if diagnoses are not detected, incorrectly recorded, or missed due to patient dropout. Indeed, most vertebral fragility fractures are clinically silent and hence not captured in electronic health records [70]. While an approach utilizing only ICD codes is potentially more comprehensive and straightforward for real-world implementation due to the quality of coverage and descriptive nature of diagnosis codes, we may miss salient clinical features captured elsewhere in the electronic health record. For example, there exist ICD codes associated with obesity, osteopenia, and osteoporosis, which represent measurements of BMI and bone mineral density on a categorical level. However, these do not reflect exact clinical measurements; the exclusion of these quantitative measurements may limit the performance and clinical impact of the algorithm. Nevertheless, it may be advantageous to utilize these ICD codes rather than the quantitative measures, as such measures in an electronic health record frequently contain human error and may not always be readily available.

In addition to data set challenges, there exist limitations inherent to assumptions of the modeling approach. The suppositions of constant time between diagnosis codes and uniform sequence length may affect performance. Exploration of more advanced methods that do not require such assumptions could improve the model and is an area of future work.

**Figure 4:**
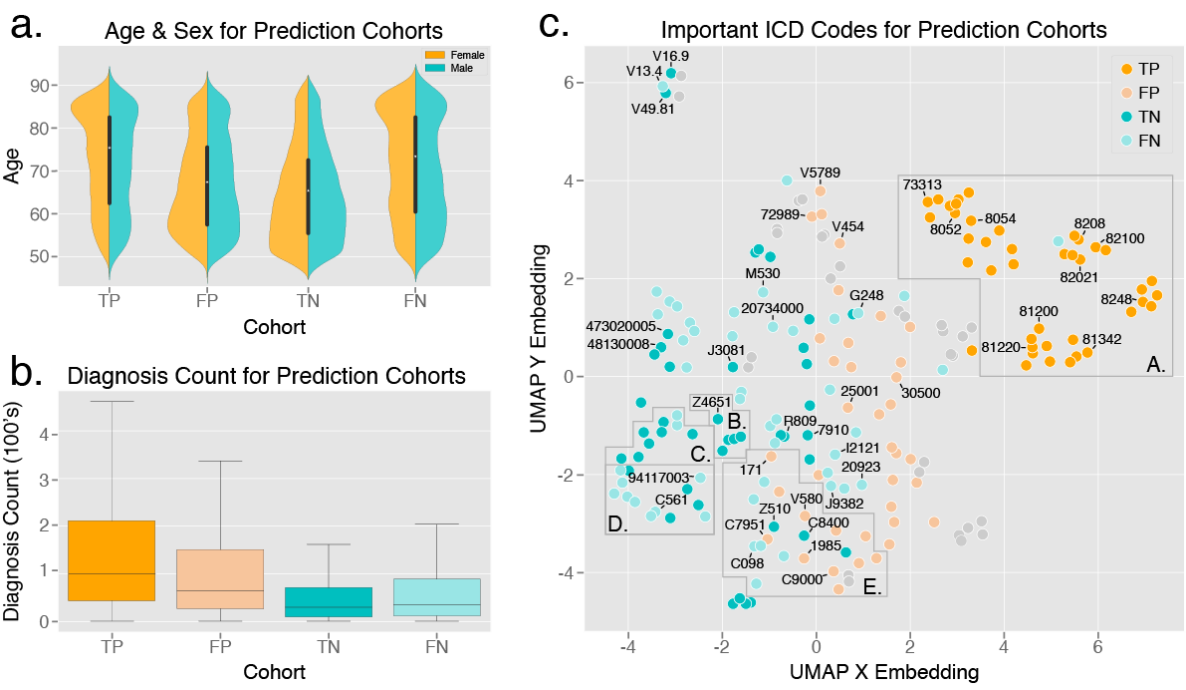
Exploration of model interpretability by comparison of various characteristics of the input data for the 4 prediction cohorts of the confusion matrix. FN: false negative; FP: false positive; ICD: International Classification of Diseases;TN: true negative; TP: true positive; UMAP: uniform manifold approximation and projection.

Perhaps the greatest limitation of the described approaches is that they are generally considered black box approaches and lack significant interpretability. Developing methods for improved interpretation of deep learning models is an active area of research. We have performed an initial exploration of this for the ICD code vectorization and long short-term memory model in Figure 4, which compares various characteristics of the four prediction cohorts of the confusion matrix for the test set (true positive [TP], false positive [FP], true negative [TN], false negative [FN]). Within each of these groups, we performed exploratory analysis on the associated samples for each of the input features in the model: age, sex, total diagnosis count, and ICD codes. Results of this analysis are described in detail in Multimedia Appendix 1. While this serves as an initial evaluation of model interpretability, a deeper exploration of interpretability techniques is an area for future work in these algorithms.

Another limitation of this study is the inability to perform direct comparisons with established risk calculators such as FRAX. Additionally, this approach has yet to be validated with external data, which is the subject of future work.

### Potential Applications

We foresee numerous applications of this work in the health care system, with benefits for patients, providers, and payers alike. For payers, Crystal Bone provides a unique opportunity to explore population health, enabling insurers to identify and address patients in need of evaluation or intervention, and preventing the large expenses associated with fracture events. For providers, direct electronic health record integration would facilitate patient care, and help identify at-risk patients who are not currently identified as such. That being said, effective implementation requires additional understanding on the impact of interventions on short-term fracture risk; while there is evidence to suggest that rapid acting treatments and bone-forming agents can significantly decrease fracture risk on a shortened time frame [8,46,47,63-69], a more detailed exploration of the optimal care pathways for various Crystal Bone risk scores would likely be required to facilitate real-world use of the algorithm.

Crystal Bone addresses the need for an automated and largely physician-independent tool that is effective at predicting short-term fracture risk. It is the first such approach that takes longitudinal patient trajectories into account, rather than focusing primarily on cross-sectional information, enabling a more personalized assessment of fracture risk. Furthermore, with automated aggregation of patient histories in an electronic health record system, the prediction of fracture risk could be entirely hands-off, without requiring a doctor or patient to manually enter any information into the software. This unique approach may facilitate broader adoption of the algorithm. Still, the lack of clinical guidelines for 1- and 2-year risk may limit adoption in the near future.

Such a tool, if widely applied, could facilitate early patient identification, and help reduce the morbidity and mortality associated with fractures. The retrospective human-level performance comparison suggests that Crystal Bone would identify patients who are currently missed in the health care system, potentially minimizing the burden on patients and the health care system overall. Given the prevalence and anticipated increase of fractures due to osteoporosis and low bone mass as the population ages, as well as the enormous personal, clinical, and economic costs associated with such fractures, Crystal Bone could provide a meaningful positive impact through reduced burden and improved outcomes.

## Appendix

Multimedia Appendix 1 Supplementary Information.

## Abbreviations

AUPRC: area under the precision-recall curve
AUROC: area under the receiver operating characteristics curve
FRAX: University of Sheffield Fracture Risk Assessment Tool
GIH-BFRC: Garvan Institute of Health Bone Fracture Risk Calculator
ICD: International Classification of Diseases
