# Supplementary material for: Deep Learning With Electronic Health Records for Short-Term Fracture Risk Identification: Crystal Bone Algorithm Development and Validation
Source: J Med Internet Res. 2020 Oct 16;22(10):e22550. doi: 10.2196/22550 (PMC7600029; doi:10.2196/22550)
Supplement: Multimedia Appendix 1 [file jmir_v22i10e22550_app1.doc]

**Supplementary Information**

**Labeling Criteria**

To identify the case and controls for this model, we defined a set of rules based on those used by Wright et al for identifying novel fracture events in claims data [1]. This set includes open and closed fractures in the vertebrae, pelvis, clavicle, humerus, radius, ulna, hip, femur, tibia, fibula, and ankle. It excludes fractures in the ribs, skull, face, fingers, hands, feet, and toes. Furthermore, this set focuses on the S, M48, M80, and M84 categories in the International Classification of Diseases, Tenth Revision (ICD-10) classification system, and corresponding categories from ICD-9. Notably, this set of rules excludes fractures resulting from neoplasms and other nebulously defined fractures, such as stress fractures, or fractures from a joint replacement operation (peri-prosthetic). These are not limited to low-trauma fractures, as we did not evaluate the trauma associated with each fracture event. We refer to this set holistically as the Wright fractures. In the panther data set, these rules identified 59,918 qualifying fracture events that met our inclusion criteria. Supplementary Figure 1 shows the distribution of fracture regions in the holdout set of the panther data, which is aligned with an analogous distribution reported in the Amin et al study, a population-based study in Olmsted County, Minnesota, which spanned 20 years and covered several thousand patients [2].


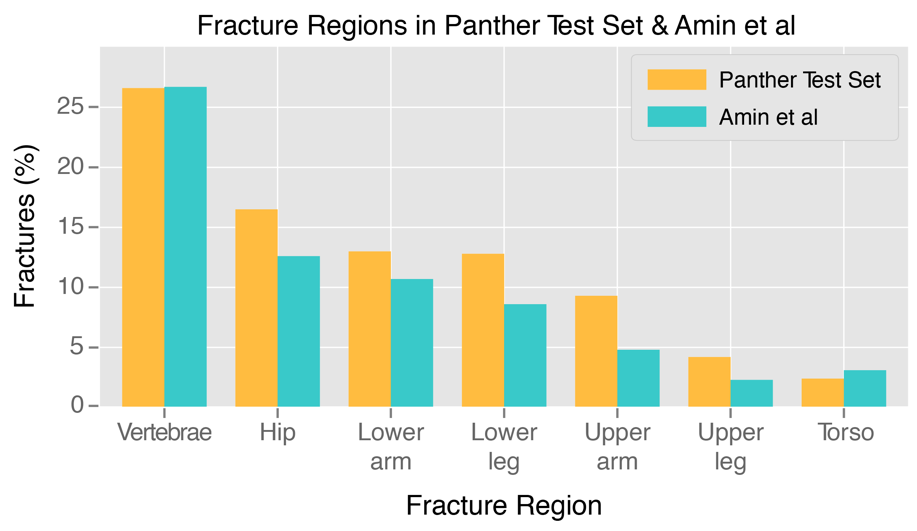


**Supplementary Figure 1.** Distribution of fracture regions. To evaluate the distribution of fracture regions identified in the panther test set, we compared our results to those of a study performed by Amin et al. We observed that the incidence rates of each fracture region were aligned with that found in the literature.

In electronic health record systems, ICD codes for events like fractures tend to be repeated frequently. As such, it can be difficult to understand exactly when a fracture occurs. We used the following set of rules adapted from Wright et al [1] to reasonably determine unique (novel) fracture events:

1. There were zero prior fracture codes before the current code, OR
2. The most recent prior fracture code occurred >90 days before the current code, OR
3. The prior fracture code occurred ≥30 days before the current code, AND is in a new region of the body.

**Sliding Window Approach**

Once the fracture and non-fracture events were labeled, we generated input data for the algorithms with a sliding window approach. Starting with the event date, we extracted the sequence of ICD codes occurring within the 2-year window leading up to this date. We then iteratively slid the window back in time by 1 year and repeated the sequence extraction process. The tail end of a sequence must be ≤2 years before a qualifying fracture event date to be considered a fracture sequence. We repeated this process up to five times, generating a maximum of five unique sequences for each event in the data. If there were not 2 years’ worth of diagnosis history for a given window, we stopped the sliding process and dropped the incomplete window from the analysis.

In patients with secondary fracture(s), over 95% of these fractures occurred within 5 years of the prior fracture. As such, the sliding window process for secondary fracture events can produce partially or completely contradicting sequence samples (sequences that qualify as both fracture and non-fracture). To minimize any unintended duplication of sequences, we chose to keep only the first three windows for each subsequent fracture event. One effect of this approach is that it may indirectly decrease the number of historical windows that contain fractures in the look-back period, but do not correspond to a positive label (fracture event) in the future. This may inflate the effect of historical fracture as a predictor of fracture risk. However, because prior fracture is well established as a strong predictor of risk [3-6], we are willing to accept this possibility; the described sliding window approach enables a 2-year time horizon while also maximizing the number of fracture events in the data, a useful approach for an imbalanced classification task such as this one. The schematic in Figure 1 of the manuscript provides a visual explanation of the sliding window process.

**Hyperparameter Tuning**

To train and optimize all model configurations (including the baselines), we performed hyperparameter tuning with a three-fold cross-validation scheme on the training data set. To do so, we utilized the Hyperopt framework, which implements a Bayesian method for optimizing hyperparameter selection [7]. We used the obtained hyperparameters to train the final model on the balanced training set and evaluated the performance of these trained models on the holdout set.

**Human-Level Performance Details and Limitations**

In the cohort analysis of the human-level performance comparison, the primary goal was to understand the relative benefit of using Crystal Bone for patient identification. We sought to evaluate how well the algorithm captures patients who are currently “missed” by their physicians, as well as how often the algorithm incorrectly flags patients who do not ultimately fracture. In order to conservatively estimate these benefits, we chose to use a broad definition of “intervention,” which includes all possible interventions (treatments, tests, diagnoses) as shown in Table 1 of the manuscript.

In the overlap analysis, the primary goal was to gauge the extent to which the algorithm flags “agree” with human-level performance interventions. A limitation of this approach is that Crystal Bone was trained for the specific task of predicting the likelihood of a fracture in 2 years, and identification of a 2-year risk is not currently a part of any physician protocol or guideline. Thus, physicians may intervene with varying interpretations of risk and time horizons, and as a result we cannot expect a perfect overlap between human-level performance and Crystal Bone. In order to more fairly compare the algorithm with human-level performance, we focused solely on treatment interventions, as these are likely more urgent than diagnostic tests, and therefore more analogous to the 2-year prediction task that is performed by the algorithm. Evaluating overlap before pharmacological interventions occur allows us to better understand how the algorithm’s performance compares to that of physicians.

The primary challenge with retrospective human-level performance analysis is that we treat observed interventions in the electronic health record as a proxy for a physician’s identification of short-term risk. There may be cases where the physician has identified a risk of fracture without performing an intervention that was captured in the data. The inverse is also true; some of these interventions may occur routinely, or in response to other unknown factors, rather than in response to perceived risk. We attempt to mitigate this through adjusting our definition of “intervention” for each experiment; however, the inability to capture human-level performance at this level of nuance remains a limitation of this approach. Nevertheless, such retrospective analysis provides scalability and avoids bias.

**Patient-Level Embeddings**

Because both ICD code vectorization and patient-level vectorization techniques are unsupervised and do not perform an immediate classification, it is difficult to characterize their performance. With the patient-level vectorization approach, this is compounded by the fact that the vectors represent a more abstract concept than the ICD code vectors. Establishing similarity between patients given their entire history of diagnoses is non-trivial. Unlike ICD codes, which come with a given definition, patient profiles have no pre-determined ground truth. Furthermore, the patient vector representations encode not only which specific ICD codes a patient has, but also the relative frequency and specific combinations of these codes. In spite of these complexities, we attempted an empirical approach to project the encoded patient vectors in two dimensions using uniform manifold approximation and projection (UMAP) and visualize patient patterns for various characteristics (Supplementary Figure 2).


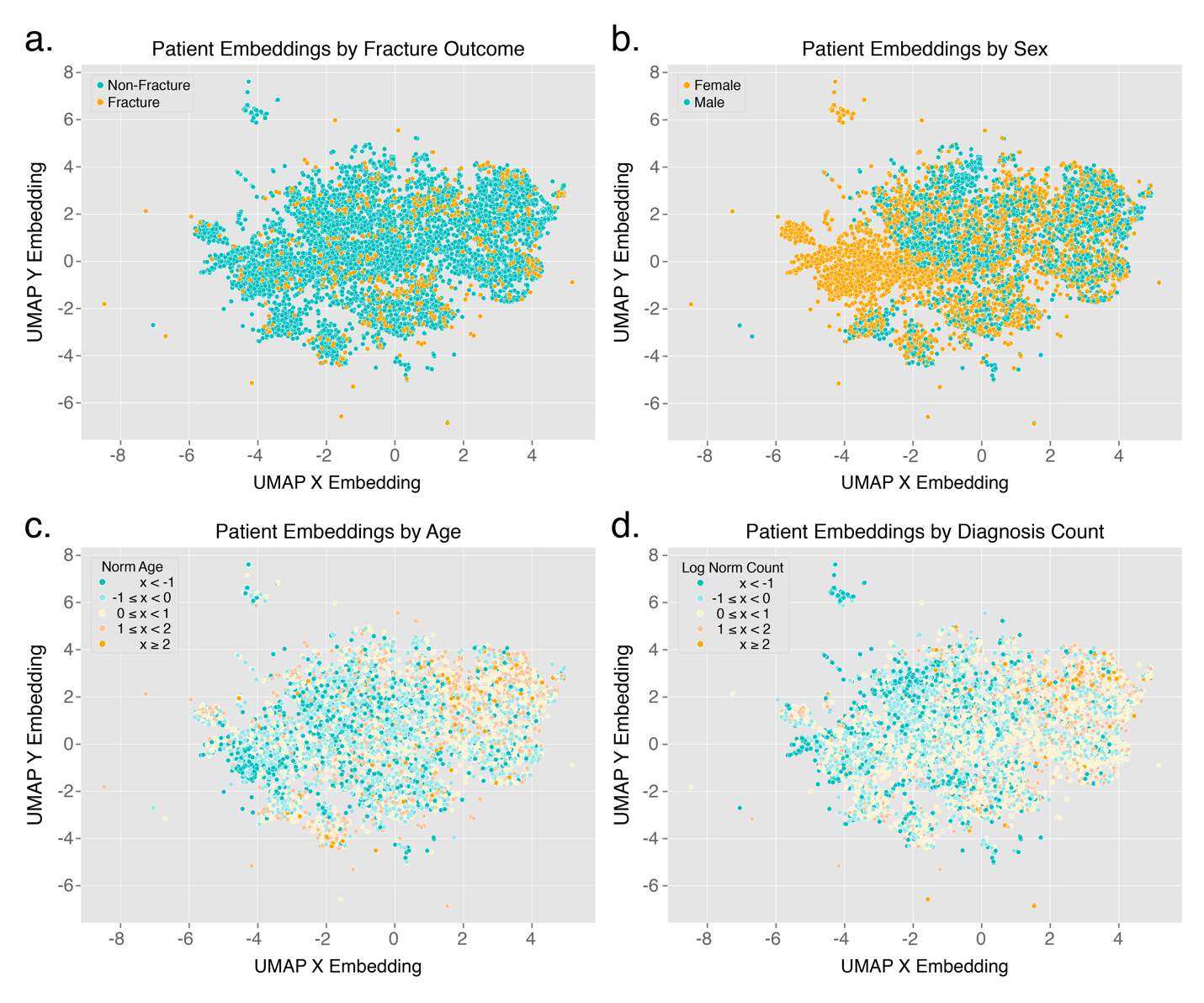


**Supplementary Figure 2.** 2D projection of the patient-level vectorization embeddings. These figures represent a random 1% sample of the testing data set from panther, colored by various characteristics for qualitative evaluation. While these embeddings appear to encode some patient characteristics, the collocation is not as clearly defined as that of the ICD code vectorization model (Figure 2). (a) Patient vectors colored by fracture outcome. While some of the fracture patients appear to cluster near each other, there does not appear to be an overall pattern across the data. (b) Patient vectors colored by sex. There is a clear cluster of women on the left side of the graph, indicating that some latent information was encoded. (c) Patient vectors colored by normalized age. There appears to be some trend along the x-axis, with age increasing as x axis increases. (d) Patient vectors colored by log normalized diagnosis count (total number of ICD codes in the patient’s sequence). As with age, there appears to be a trend that increases along the x-axis, and potentially shares some relationship with age. We hypothesize that higher diagnosis count is associated with worse overall health (more codes reflect more illnesses or conditions), thus it follows that there may be a relationship with age. Again, we have some qualitative evidence that latent information regarding the patient journey has been encoded through the patient-level vectorization model. ICD=International Classification of Diseases; UMAP=uniform manifold approximation and projection**.**

We experimented with several coloring schemes in the figures to evaluate patient similarity. In light of the aforementioned limitations, evaluating these plots by the collocation of specific characteristics is an imperfect method to judge the performance of this method. This validation technique is itself abstract, and ultimately it is impossible to assess the correctness of the patient embeddings with the same degree of confidence that we have in the ICD code embeddings.

**Framework 1: Primary vs Subsequent Fractures**

We chose to perform additional analyses with the ICD code vectorization and long short-term memory networks framework because it had the best overall performance with respect to area under the receiving operating characteristics curve (AUROC), as observed in Table 1 of the manuscript. An additional benefit to focusing on this approach is its relative interpretability; it is more straightforward to extract insights from this framework as the inputs to the model represent individual ICD codes.

To understand the model behavior better, we compared performance on primary and subsequent fracture events. We explored this analysis for several reasons. Firstly, because of the difficulty in de-duplicating fracture events, it is challenging to confirm if subsequent fracture events are novel, rather than repeated codes reflecting historical events. Thus, we were curious to see the model’s performance on primary fractures specifically, as these are the most confidently defined fracture events in the data (though there is still some uncertainty due to the coverage of the data set, which starts in 2007). Furthermore, we wanted to evaluate whether the model is better suited to predict primary fractures compared with subsequent fractures. Finally, we sought to better evaluate the effect of prior fracture events on the quality of the predictions, through comparison of the performance of primary and subsequent fracture models. To perform this analysis, we built distinct models for predicting primary and subsequent fractures, rather than performing sensitivity analysis on the all-fracture model. To our knowledge, this is the first study that has experimented with a separate model for each of these fracture types.

The separate primary and subsequent fracture models were trained by the same paradigm as the all-fracture model; each model utilized a data set that was modified to exclusively contain either primary or subsequent fracture types. For these models, the oversampling of corresponding fracture events from the osteo data set still occurs in the training data set, and the holdout set is also untouched.

Each model saw only the fractures from one type (primary, subsequent). Both the primary and subsequent fracture model also underwent hyperparameter tuning with a 3-fold cross-validation scheme, resulting in model architectures that are slightly different across the three experiments. Supplementary Table 1 shows the performance metrics on the unique holdout set for each distinct model for both the ICD code vectorization and long short-term memory network and the baseline (age + sex + diagnosis count) frameworks.

**Supplementary Table 1.** Analysis of primary vs subsequent fracture

|  |  | AUROC | Recall | Specificity | Precision | AUPRC |
| --- | --- | --- | --- | --- | --- | --- |
| **Crystal Bone** | All Fx | 0.812 | 0.646 | 0.812 | 0.192 | 0.462 |
|  | Primary Fx | 0.742 | 0.517 | 0.812 | 0.0999 | 0.160 |
|  | Subsequent Fx | 0.910 | 0.683 | 0.962 | 0.342 | 0.676 |
| **Baseline** | All Fx | 0.668 | 0.547 | 0.707 | 0.114 | 0.130 |
|  | Primary Fx | 0.591 | 0.126 | 0.934 | 0.0715 | 0.0549 |
|  | Subsequent Fx | 0.747 | 0.300 | 0.932 | 0.113 | 0.0898 |

We compared performance metrics for a 2-year horizon when predicting all, primary, and subsequent fractures with the ICD code vectorization and long short-term memory framework, showing the respective performance of the baseline approach (using age, sex, and diagnosis count) for each fracture type. For this framework, the performance of the subsequent fracture model is substantially better than those of the other two models, showing improvement across all performance metrics. For the baseline analyses, the AUROC of the subsequent model is improved over the other two, and the specificity of the subsequent model is improved over that of the all-fracture model, but all other performance metrics of the subsequent fracture baseline decrease when compared with the all-fracture baseline. Each Crystal Bone model shows a marked improvement in performance when compared with its respective baseline across nearly all metrics. AUPRC=area under the precision-recall curve; AUROC=area under the receiving operating characteristics curve; Fx=fracture.

It is evident that the ICD code vectorization and long short-term memory framework’s performance on subsequent fracture prediction is more accurate than its performance on primary fractures. The metrics associated with predicting subsequent fracture are quite high, reflecting a model with significant predictive power. These results suggest that in general, subsequent fracture events are easier to predict.

The human-level performance analysis supports these findings. In the cohort human-level performance analysis, we saw Crystal Bone identified 56.3% of the non-intervention windows that ultimately fractured. When broken down into primary and subsequent fracture types, this represented 48.9% capture for primary fractures, and 77.1% capture for subsequent fractures. Additionally, for the intervention cohort from this analysis, Crystal Bone flagged 83.9% of the windows that fractured within 2 years, consisting of 74.2% and 93.5% capture for primary and subsequent fracture, respectively. It is possible that this improved performance is because the algorithm recognized that a historical fracture in the look-back period is a risk factor for future fracture events, a relationship that has been well-documented in the literature [3-6].

While performance for subsequent fractures is better overall, there is a substantial improvement in the performance of both models as compared with their respective baselines. The primary fracture AUROC improved by 0.151, and the subsequent fracture AUROC improved by 0.163. These results, paired with the primary fracture AUROC of 0.742, indicate that Crystal Bone is still able to produce an effective model for the prediction of primary fracture events, without relying on evidence of prior fracture in the historical input sequences. Additionally, these findings suggest that regardless of fracture type, incorporating longitudinal information in the prediction results in an improved performance overall.

**Fracture Region Analysis**

In order to further understand the ICD code vectorization and long short-term memory model’s performance, we performed a subgroup analyses, in which we evaluated performance changes across fracture regions. To do so, we used the seven region labels defined by the Wright rules: vertebrae, hip, lower leg, lower arm, upper arm, upper leg, and torso. Each fracture event is labeled based on the set of regions associated with it, ergo some fracture events involve fractures in more than one region of the body. For each of these regions, we evaluate the performance of the model in terms of sensitivity, or recall, based on fractures associated with the region. In general, it appears that the model performs comparably well across all regions, with some slight bias toward the regions that appear more frequently in the data (Supplementary Table 2).

**Supplementary Table 2.** Fracture region sensitivity analysis

|  | Vertebrae | Hip | Upper leg | Lower arm | Upper arm | Lower leg | Torso |
| --- | --- | --- | --- | --- | --- | --- | --- |
| Recall | 0.655 | 0.585 | 0.612 | 0.531 | 0.537 | 0.567 | 0.572 |

Comparison of the Wright fracture rules, 2-year horizon, all-fracture model’s sensitivity across each of the seven regions. The model performs slightly better for vertebrae and femur fractures; however, overall sensitivity is fairly consistent across regions.

This subgroup analysis suggests that the model does not have explicit bias toward fractures in any one region of the body, despite the stochasticity that may be associated with such scenarios.

**Model Robustness**

To further analyze the performance of the ICD code vectorization and long short-term memory model, we evaluate its robustness to both the time horizon for the prediction and the definition of fracture used to label the ground truth. First, we compare the performance of the model using a 1-year horizon for fracture, rather than a 2‑year horizon. To generate the model, all of the data engineering outside of the sliding window labels remain constant. The only modification is that the edge of the window must be less than or equal to 1 year before the fracture for the sequence to be a “fracture window.” When comparing the performance of this model on a 1-year horizon with that on a 2-year horizon, we see general consistency in the overall performance metrics.

**Supplementary Table 3. Analysis of 1-year vs 2-year risk horizon**

|  | AUROC | Recall | Specificity | Precision | AUPRC |
| --- | --- | --- | --- | --- | --- |
| 2-year horizon | 0.812 | 0.646 | 0.812 | 0.192 | 0.462 |
| 1-year horizon | 0.819 | 0.634 | 0.835 | 0.163 | 0.455 |

We compared the performance metrics for the ICD code vectorization and long short-term memory model with a 2-year horizon to those of the model with a 1-year horizon. The performance is fairly consistent across all five metrics. AUPRC=area under the precision-recall curve; AUROC=area under the receiving operating characteristics curve.

Additional validation of model robustness comes from adjusting the definition of fracture. In the Methods section of the manuscript, we outlined the Wright rules for defining fracture. In addition to these rules, we also developed a more liberal set of rules which we refer to as the DHI rules (named for Digital Health & Innovation, the team at Amgen Inc. that wrote this study). In addition to all Wright fractures, the DHI fractures also include rib, hand, and foot fractures. Furthermore, the DHI rules also include fractures resulting from neoplasms and other “edge case” fractures, such as stress fractures or fractures from a joint replacement operation. Supplementary Table 4 shows the results of this fracture rule comparison.

**Supplementary Table 4. Analysis of Wright vs DHI fracture definitions**

|  | AUROC | Recall | Specificity | Precision | AUPRC |
| --- | --- | --- | --- | --- | --- |
| Wright fractures | 0.812 | 0.646 | 0.812 | 0.192 | 0.462 |
| DHI fractures | 0.794 | 0.618 | 0.811 | 0.253 | 0.483 |

Comparison of the ICD code vectorization and long short-term memory model performance metrics with the Wright fracture rules, to those of the model with the DHI fracture rules. Both models utilized a 2-year risk horizon. Again, we see consistent performance across both analyses. AUPRC=area under the precision-recall curve; AUROC=area under the receiving operating characteristics curve; DHI=Digital Health & Innovation; LSTM=long short-term memory.

Again, the performance is largely maintained. There is a slight decline in performance overall, which is likely due to the incorporation of potentially “non-osteoporotic” fractures that comes with the more liberal DHI fracture rules. These evaluations, with a modified time horizon as well as a modified definition of fracture, reflect that the ICD code vectorization and long short-term memory framework is fairly robust to modifications in experiment design and support the generalizability of the model.

**ICD Code Vectorization and Long Short-Term Memory: Exploring Interpretability**

Although there is no straightforward way to extract feature importance from either model approach presented here, we performed error analyses on the ICD code vectorization and long short-term memory model to gain some insight with respect to model interpretability. The error analysis consisted of splitting the predictions into each category of the confusion matrix: true positives (TP), true negatives (TN), false positives (FP), and false negatives (FN). For each of these groups, we performed exploratory analysis on the associated samples. We began by evaluating the static features of age, sex, and diagnosis count. For each group, we generated distributions of these features to characterize any noticeable differences that may contribute to the prediction. These results are shown in Figure 4a and 4b of the manuscript.

In Figure 4a, there appears to be a trend in the age + sex distribution based on the actual label rather than the predicted label. This is counterintuitive; if the model’s prediction is influenced by age and sex, we would expect the prediction cohorts’ distributions of age and sex to be aligned based on the predicted result (ie, TP ~ FP and TN ~ FN); however, we see the opposite behavior. This may suggest that the model relies more heavily on diagnosis count and sequence information than it does on age and sex. Such a conclusion is augmented by Figure 4b, in which the distribution for diagnosis codes are aligned by the predicted label, as expected. There appears to be a direct relationship between diagnosis count and directionality of the model prediction. This observed trend suggests that a higher overall diagnosis count pushes the model toward a positive prediction.

In addition to analyzing the static features across these subgroups, we also explored the values associated with the sequential data. Because the model learns to recognize patterns and frequencies in addition to specific codes, it is impossible to fully characterize how the model interprets the ICD code inputs. In spite of this, we attempted to do so by evaluating the frequently relevant codes in each subgroup.

Recall that the ICD code embeddings encode the relationships between similar codes. As such, to understand which types of codes commonly occur in each group, we can return to a two-dimensional UMAP embedding to evaluate clusters of codes. We use term frequency – inverse document frequency (TF-IDF) [9] to identify the 50 most important codes for each prediction cohort (TP, TN, FP, FN). Each cohort is treated as a document when calculating this statistic, and the method identifies relevant terms by assessing their relative frequency across the cohorts (eg, high frequency in document 1 with low frequency across all other documents corresponds to a relevant term for document 1). We then use UMAP to visualize these top 200 relevant points in two dimensions, using the clusters in the projection to better understand trends in the ICD code profiles of each group (shown in Figure 4c of the manuscript).

As expected, there is some overlap between the codes for the four groups. However, as with the diagnosis count feature, there appears to be a trend based on the predicted label, with positive prediction cohorts on the right side of Figure 4c and negative prediction cohorts on the left. Interestingly, there appears to be strong segmentation among the ICD codes associated with TP, indicating that the model has learned some concrete characteristics for this subgroup. Region A in Figure 4c, which contains the majority of these TP, is almost entirely made up of fracture codes. This suggests that evidence of a prior fracture will push the model toward a positive prediction, a relationship that has been validated extensively in the literature [3-6].

There is also some collocation among the negatively predicted groups. In particular, region B contains codes associated with being obese or overweight. This region exclusively contains TN, reflecting an association between high BMI and protection from osteoporotic fracture that has been validated in the literature [10, 11]. We also see that region C, which consists of codes found only in pre-menopausal women (ie, pregnancy test, birth control, menstrual cramps, etc) also consists largely of TN. This suggests that the model recognizes a link between menopause and osteoporosis; namely that pre-menopausal women are at lower risk [12-14].

Ultimately, such trends within these subgroups may explain some of the correct predictions as well as the misclassifications made by the model. Although it is impossible to confirm how exactly the model utilizes these features to inform its prediction, the patterns in these codes offer some interpretability for the approach.

**References**

1. Wright NC, Daigle SG, Melton ME, Delzell ES, Balasubramanian A, Curtis JR. The design and validation of a new algorithm to identify incident fractures in administrative claims data. J Bone Miner Res 2019;34(10):1798–1807. doi: 10.1002/jbmr.3807. PMID: 31170317.

2. Amin S, Achenbach SJ, Atkinson EJ, Khosla S, Melton LJ. Trends in fracture incidence: a population-based study over 20 years. J Bone Miner Res 2014;29(3):581–589. doi: 10.1002/jbmr.2072. PMID: 23959594.

3. Bliuc D, Nguyen ND, Nguyen TV, Eisman JA, Center JR. Compound risk of high mortality following osteoporotic fracture and refracture in elderly women and men. J Bone Miner Res 2013;28(11):2317–2324. doi: 10.1002/jbmr.1968. PMID: 23616397.

4. Center JR, Bliuc D, Nguyen TV, Eisman JA. Risk of subsequent fracture after low-trauma fracture in men and women. JAMA 2007;297(4):387–394. doi: 10.1001/jama.297.4.387. PMID: 17244835.

5. Kanis JA, Johnell O, De Laet C, Johansson H, Oden A, Delmas P, et al. A meta-analysis of previous fracture and subsequent fracture risk. Bone 2004;35(2):375–382. doi: 10.1016/j.bone.2004.03.024. PMID: 15268886.

6. Gehlbach S, Saag KG, Adachi JD, Hooven FH, Flahive J, Boonen S, et al. Previous fractures at multiple sites increase the risk for subsequent fractures: the Global Longitudinal Study of Osteoporosis in Women. J Bone Miner Res 2012;27(3):645–653. doi: 10.1002/jbmr.1476. PMID: 22113888.

7. Komer B, Bergstra J, Eliasmith C. Hyperopt-sklearn: automatic hyperparameter configuration for Scikit-learn. 13th Python in Science Conference 2014. 32–37.

8. Le QV, Mikolov T. Distributed representations of sentences and documents. 2014:arXiv:1405.4053 [cs].

9. Ramos J. Using TF-IDF to determine word relevance in document queries. 2003. Paper presented at the proceedings of the first instructional conference on machine learning, Piscataway, NJ. <https://sites.google.com/site/caonmsu/ir/UsingTFIDFtoDetermineWordRelevanceinDocumentQueries.pdf>.

10. Shen J, Leslie WD, Nielson CM, Majumdar SR, Morin SN, Orwoll ES. Associations of body mass index with incident fractures and hip structural parameters in a large canadian cohort. J Clin Endocrinol Metab 2016;101(2):476–484. doi: 10.1210/jc.2015-3123. PMID: 26670128.

11. Johansson H, Kanis JA, Odén A, McCloskey E, Chapurlat RD, Christiansen C, et al. A meta-analysis of the association of fracture risk and body mass index in women. J Bone Miner Res 2014;29(1):223–233. doi: 10.1002/jbmr.2017. PMID: 23775829.

12. Kanis JA, Pitt FA. Epidemiology of osteoporosis. Bone 1992;13:S7–S15. doi: 10.1016/S8756-3282(09)80004-5. PMID: 1581122.

13. Lerner UH. Bone remodeling in post-menopausal osteoporosis. J Dent Res 2006;85(7):584–595. doi: 10.1177/154405910608500703. PMID: 16798857.

14. Lindsay R. The menopause and osteoporosis. Obstet Gynecol 1996;87(2 Suppl):16S–19S. doi: 10.1016/0029-7844(95)00430-0. PMID: 8559548.
